# Effects of Primary Processing Techniques and Significance of Hall-Petch Strengthening on the Mechanical Response of Magnesium Matrix Composites Containing TiO_2_ Nanoparticulates

**DOI:** 10.3390/nano5031256

**Published:** 2015-07-31

**Authors:** Ganesh Kumar Meenashisundaram, Mui Hoon Nai, Manoj Gupta

**Affiliations:** Department of Mechanical Engineering, National University of Singapore, 9 Engineering Drive 1, Singapore 117576, Singapore; E-Mails: ganesh_kumar@u.nus.edu (G.K.M.); mbinmhb@nus.edu.sg (M.H.N.)

**Keywords:** Mg (1.98 and 2.5) vol % TiO_2_ nanocomposites, synthesis techniques, Hall-Petch mechanism, tensile properties, compression properties

## Abstract

In the present study, Mg (1.98 and 2.5) vol % TiO_2_ nanocomposites are primarily synthesized utilizing solid-phase blend-press-sinter powder metallurgy (PM) technique and liquid-phase disintegrated melt deposition technique (DMD) followed by hot extrusion. Microstructural characterization of the synthesized Mg-TiO_2_ nanocomposites indicated significant grain refinement with DMD synthesized Mg nanocomposites exhibiting as high as ~47% for 2.5 vol % TiO_2_ NPs addition. X-ray diffraction studies indicated that texture randomization of pure Mg depends not only on the critical amount of TiO_2_ NPs added to the Mg matrix but also on the adopted synthesis methodology. Irrespective of the processing technique, theoretically predicted tensile yield strength of Mg-TiO_2_ nanocomposites was found to be primarily governed by Hall-Petch mechanism. Among the synthesized Mg materials, solid-phase synthesized Mg 1.98 vol % TiO_2_ nanocomposite exhibited a maximum tensile fracture strain of ~14.5%. Further, the liquid-phase synthesized Mg-TiO_2_ nanocomposites exhibited higher tensile and compression properties than those primarily processed by solid-phase synthesis. The tensile-compression asymmetry values of the synthesized Mg-TiO_2_ nanocomposite was found to be lower than that of pure Mg with solid-phase synthesized Mg 1.98 vol % TiO_2_ nanocomposite exhibiting as low as 1.06.

## 1. Introduction

Magnesium (Mg) is the lightest of all structural metals having a low density of 1.74 g/cm^3^ which is approximately two-thirds that of Al (2.7 g/cm^3^), one-fifth that of steel (7.9 g/cm^3^) and in close comparison to that of plastics (0.92–2.17 g/cm^3^) [1]. Contributing to approximately 2.7% by weight, Mg is the seventh most abundant element in the earth’s crust. Besides being light, Mg based materials also exhibit other important properties such as good castability, machinability, thermal stability, good damping characteristics, high specific mechanical properties and resistance to electromagnetic radiation [2,3,4]. Among the other metals, pure iron and Mg have been reported to possess excellent biocompatibility with no signs of systemic or local toxicity [5,6,7]. In the past ten years, number of scientific publications on magnesium as a “biomaterial” has exponentially increased [8]. The recommended daily intake of magnesium for human beings is ~240 to 420 mg/day which is ~17 to 50 times more than that of iron (~8 to 18 mg/day) [9]. Further, when compared to other metals, the Young’s modulus of Mg materials (40–45 GPa) is closer to that of natural bone (3–20 GPa) and thereby assists in mitigation of stress shielding effects with possibility to eliminate secondary surgery for the implant removal when utilized as a biomaterial especially for orthopedic applications. However, its poor ductility at room temperature restricts its extensive applications. Presence of possible deformation mechanisms such as basal slip, prismatic slip, pyramidal slip and several twinning modes complicates the deformation behavior of materials with hexagonal close-packed (HCP) crystal structure such as Mg [10].

To realize simultaneous improvements in both strength and ductility of magnesium materials, when compared to alloying and grain refinement, dispersion strengthening through addition of inexpensive nanoparticulates (NPs) is getting tremendous attention and without noticeable weight gains of Mg materials, improvements through nanocomposite approach is possible and thereby considered as a promising technique [11]. The major mechanisms contributing to the strengthening of particulate reinforced metal matrix nanocomposites (MMNCs) are [11]: (1) Orowan strengthening from dislocation bowing by NPs; (2) Hall-Petch strengthening from grain refinement; (3) Forest strengthening resulting from mismatch in the coefficient of thermal expansion values (CTE) between Mg matrix and NPs; (4) Taylor strengthening due to mismatch in the modulus values between the Mg matrix and NPs; and (5) strengthening due to load bearing of NPs. For simultaneous improvement in the strength and ductility of MMNCs, activation of Orowan strengthening mechanism is critical since the ductility of MMNCs is not compromised during the strengthening when compared to strengthening by Forest and Taylor mechanisms [11]. Orowan strengthening depends on the following factors: (a) size of the NPs, (b) quantity of the NPs and (c) interparticulate spacing between the NPs within the metal matrix. Apart from the size and quantity of the NPs, interparticulate spacing is greatly controlled by the type of synthesis technique utilized to uniformly disperse the NPs within the metal matrix. The synthesis techniques or processing methods for fabrication of Mg composites can be classified into the following categories [1]: (1) liquid-phase methods where the particulate reinforcements are added into liquid Mg metal matrix and (2) solid-phase methods where the syntheses of composites are done at a temperature below the solidus temperature of the Mg metal matrix phase. Cost effective solidification processes which enable efficient dispersion of NPs and blend-press-sinter powder metallurgy techniques are essentially important liquid and solid phase processing routes, respectively, for synthesizing high performance Mg MMNCs.

Further, natural bone is a bio-“nanocomposite” made up of collagen fiber matrix and hydroxyapatite crystals [12]. Owing to the similarities between Mg MMNCs and natural bone as “nanocomposites”, selection of ultrafine particulates as reinforcement to magnesium matrix is of prime importance targeting implant applications. Titania (TiO_2_) is a bioactive material and a preferred reinforcement to improve bioactivity of composite materials. It is also used for surface treatment of metallic and ceramic implants to improve bioactivity [13]. It has high mechanical resistance, biocompatibility, chemical stability in aqueous environments, chemical inertness and comes at low cost [14,15,16,17]. Aquatic toxicity of nano TiO_2_ exhibited no or low toxicity to aquatic organisms [18]. The results of the literature search, however, reveal that no attempt is made till date to study the effects of primary processing techniques such as DMD or disintegrated melt deposition (liquid-phase synthesis) and PM or powder metallurgy technique (solid-phase synthesis) on the microstructure and mechanical properties of TiO_2_ reinforced Mg nanocomposites.

Accordingly, in the present study, Mg matrix reinforced with (1.98 and 2.5) vol % TiO_2_ NPs are primarily synthesized utilizing blend-press-sinter powder metallurgy technique. The hot extruded nanocomposites were then characterized for their physical, microstructural and mechanical properties and compared to that of DMD synthesized Mg-TiO_2_ nanocomposites. Particular emphasis of this study is to analyze the effects of synthesis on the interparticulate spacing between the NPs within the Mg metal matrix. Using the experimentally observed interparticulate spacing between the NPs, the actual contribution of Orowan strengthening to the theoretically predicted tensile yield strength (TYS) of Mg-TiO_2_ nanocomposite is ascertained. The major strengthening contributions to the TYS of the Mg-TiO_2_ nanocomposite synthesized by both DMD and PM techniques are compared and analyzed. The TYS of the synthesized nanocomposites are theoretically predicted using the existing mathematical models in the literature and are compared to that of the experimental 0.2% TYS values.

## 2. Results

### 2.1. Density and Porosity Measurements

The experimental density (ρ_exp_) of Mg materials measured by utilizing Archimedes principle (Table 1) was found to be closer to that of their calculated theoretical density (ρ_theo_). With addition of TiO_2_ NPs, only a marginal increase in the density values of pure Mg was observed and Mg 2.5 vol % TiO_2_ nanocomposite synthesized by DMD technique exhibited a maximum experimental density of ~1.8 g/cm^3^ which is ~3.5% greater than that of pure Mg (1.74 g/cm^3^). The porosity value of pure Mg was found to increase with the addition of TiO_2_ reinforcements and among the synthesized Mg materials, the PM (solid-phase) processed materials were found to possess higher porosity with Mg 2.5 vol % TiO_2_ nanocomposites exhibiting the maximum of ~0.3%.

**Table 1:** Density and coefficient of thermal expansion values (CTE) measurements of pure magnesium and synthesized Mg-TiO_2_ nanocomposites.

Material	Synthesis	Reinforcement		Density Measurements			CTE (× 10^−6^ /K)
		wt %	vol %	Theoretical ρ_theo_ (g/cm^3^)	Experimental ρ_exp_ (g/cm^3^)	Porosity (%)	
Pure Mg	DMD	Nil		1.7400	1.7380	0.1150	27.00
	PM				1.7377	0.1322	24.72
Mg 1.98 TiO_2_	DMD	4.67	1.98	1.7923	1.7895	0.1563	26.60
	PM				1.7880	0.2400	24.53
Mg 2.5 TiO_2_	DMD	5.87	2.50	1.8023	1.7990	0.1832	26.50
	PM				1.7965	0.3220	24.42

### 2.2. X-Ray Diffraction (XRD) Studies

**Figure 1:**
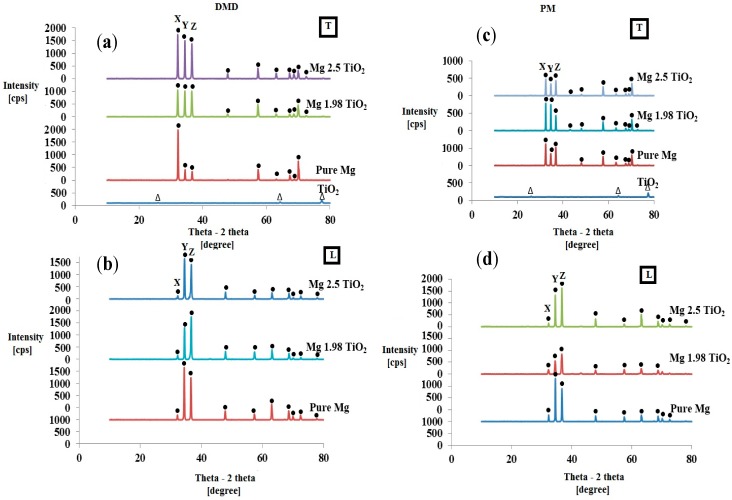
X-Ray diffractograms of TiO_2_ nanopowder, pure Mg and Mg-TiO_2_ nanocomposites synthesized by DMD (**a**) and (**b**) and PM (**c**) and (**d**) techniques taken: along transverse direction (**a)** and (**c**) and along the longitudinal direction (**b**) and (**d**) of the hot extruded samples. *X*, *Y*, *Z* represent 2θ = 32°, 34° and 36° corresponding to (10-10) prism, (0002) basal and (10-11) pyramidal planes, respectively.

Figure 1 shows the X-ray diffractograms of TiO_2_ nanopowder and Mg-TiO_2_ nanocomposite samples synthesized utilizing both DMD and PM techniques obtained from the transverse and longitudinal sections of the hot extruded samples. The high intensity Mg peaks were prominently seen and the peaks corresponding to TiO_2_ were not visible in the synthesized Mg-TiO_2_ nanocomposites which is due to the limitation of filtered X-ray to detect phases with low volume fraction [19]. However, the presence of TiO_2_ NPs in both DMD and PM synthesized Mg-TiO_2_ nanocomposites can be confirmed through microstructural characterization. Similar results were observed with the XRD studies of DMD synthesized Mg-TiC [20], Mg-TiB_2_ [21] and PM synthesized Mg-Y_2_O_3_ [22,23] nanocomposites.

With the addition of TiO_2_ NPs, changes in the basal plane intensity of pure Mg were clearly observed. The ratio of maximum XRD intensity to the respective prismatic (10-10), basal (0002) and pyramidal (10-11) intensities of synthesized pure Mg and Mg-TiO_2_ nanocomposites indicated as *X*, *Y* and *Z* at 2θ = 32°, 34° and 36°, respectively (in Figure 1), taken along both the transverse and longitudinal sections of hot extruded samples (prior to tensile and compression testing) is shown in Table 2. Along the transverse section (perpendicular to the extrusion direction), the intensity corresponding to the basal plane of pure Mg observed with the DMD and PM synthesized Mg nanocomposite samples was found to be higher than that of respective pure Mg samples and this is due to the presence of ultrafine TiO_2_ reinforcements. Further, the intensity corresponding to prismatic plane (2θ = 32°) was found to be the maximum for all the synthesized Mg materials.

Along the longitudinal section (parallel to the extrusion direction), the XRD of hot extruded pure Mg exhibits strong basal texture having maximum intensity corresponding to the basal plane (at 2θ = 34°). Irrespective of the synthesis technique, with 1.98 vol % TiO_2_ NPs addition, the intensity corresponding to the basal plane of pure Mg was found to decrease and PM synthesized Mg 1.98 vol % TiO_2_ nanocomposite exhibited *I*_basal_/*I*_max_ value as low as 0.655. With further addition of NPs (2.5 vol % TiO_2_), irrespective of the synthesis methodologies utilized in this study, the dominance of basal plane intensity of pure Mg was observed exhibiting *I*_basal_/*I*_max_ values of 1 and 0.844 in the case of DMD and PM synthesized Mg 2.5 vol % TiO_2_ nanocomposites, respectively.

**Table 2:** X-Ray diffractogram results of as extruded Mg-TiO_2_ nanocomposites synthesized by DMD and PM techniques.

Material	Synthesis	Section	Plane	*I*/*I*_max_
Pure Mg	DMD	T	10-10 Prism	1.000
			0002 Basal	0.210
			10-11 Pyramidal	0.170
		L	10-10 Prism	0.086
			0002 Basal	1.000
			10-11 Pyramidal	0.801
	PM	T	10-10 Prism	1.000
			0002 Basal	0.545
			10-11 Pyramidal	0.838
		L	10-10 Prism	0.153
			0002 Basal	1.000
			10-11 Pyramidal	0.766
Mg 1.98 TiO_2_	DMD	T	10-10 Prism	1.000
			0002 Basal	0.962
			10-11 Pyramidal	0.958
		L	10-10 Prism	0.090
			0002 Basal	0.751
			10-11 Pyramidal	1.000
	PM	T	10-10 Prism	1.000
			0002 Basal	0.952
			10-11 Pyramidal	0.539
		L	10-10 Prism	0.214
			0002 Basal	0.655
			10-11 Pyramidal	1.000
Mg 2.5 TiO_2_	DMD	T	10-10 Prism	1.000
			0002 Basal	0.862
			10-11 Pyramidal	0.793
		L	10-10 Prism	0.085
			0002 Basal	1.000
			10-11 Pyramidal	0.962
	PM	T	10-10 Prism	1.000
			0002 Basal	0.725
			10-11 Pyramidal	0.924
		L	10-10 Prism	0.081
			0002 Basal	0.844
			10-11 Pyramidal	1.000

Notes: T and L represents XRD taken along transverse and longitudinal sections of Mg-TiO_2_ samples; *I*_max_ is the maximum XRD intensity from either prism, basal and pyramidal plane; *I* is the XRD intensity from prismatic, basal and pyramidal plane of pure Mg and the intensity corresponding to the basal plane of pure Mg is given by *I*_basal_.

### 2.3. Microstructure

**Figure 2:**
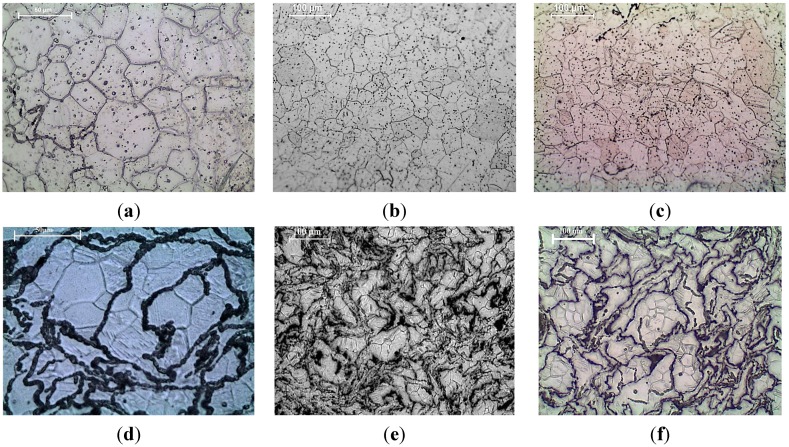
Microscopic images showing grain characteristics of: DMD synthesized (**a**) Pure Magnesium, (**b**) Mg 1.98 TiO_2_, (**c**) Mg 2.5 TiO_2_ and PM synthesized (**d**) Pure Mg, (**e**) Mg 1.98 TiO_2_, (**f**) Mg 2.5 TiO_2_.

**Figure 3:**
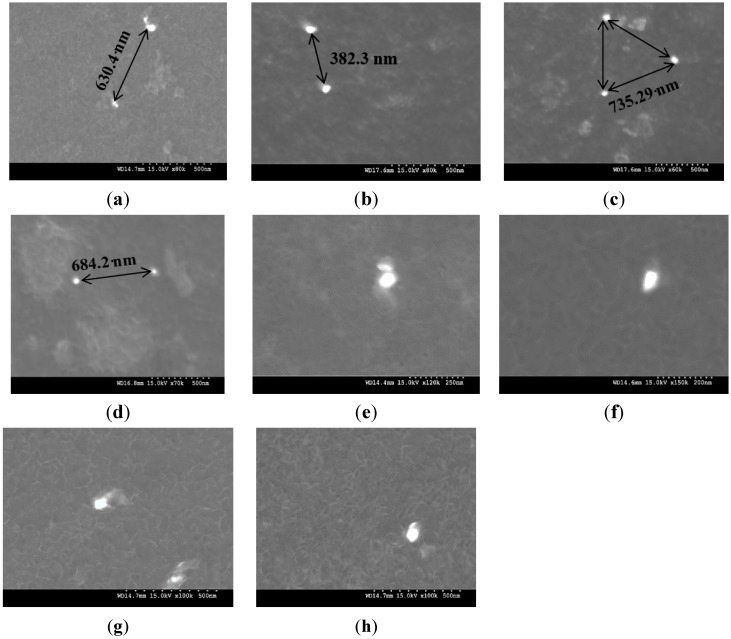
Distribution of TiO_2_ nanoparticulates in Mg-TiO_2_ composites: DMD synthesized (**a**) Mg 1.98 TiO_2_; (**b**) Mg 2.5 TiO_2_ and PM synthesized; (**c**) Mg 1.98 TiO_2_; (**d**) Mg 2.5 TiO_2_ and interfacial integrity of Mg-TiO_2_ in the case of DMD synthesized; (**e**) Mg 1.98 TiO_2_; (**f**) Mg 2.5 TiO_2_ and PM synthesized (**g**) Mg 1.98 TiO_2_; (**h**) Mg 2.5 TiO_2_.

The results of grain size measurements conducted on the optical micrographs of DMD and PM synthesized pure Mg and Mg (1.98 and 2.5) vol % TiO_2_ nanocomposites (Figure 2) are shown in Table 3. The grain size of both DMD and PM synthesized hot extruded pure Mg decreased with the addition of TiO_2_ NPs. Among the synthesized Mg materials, DMD processed Mg 2.5 vol % TiO_2_ nanocomposite exhibited the lowest grain size of ~21 μm which is ~47% lower than that of pure Mg. The minimum interparticulate spacing (λ) observed between the TiO_2_ NPs within the Mg metal matrix and their representative micrographs are shown in Table 4 and Figure 3, respectively. It is observed that the measured value of λ for DMD synthesized Mg (1.98 and 2.5) vol % TiO_2_ nanocomposites was found to be less than that of respective PM synthesized Mg-TiO_2_ nanocomposites.

**Table 3:** Results of microstructure and microhardness studies.

Sl. No.	Material	Synthesis	Grain Size (× 10^−6^ m)	Aspect Ratio	Microhardness (Hv)
1	Pure Mg	DMD [24]	45 ± 2.4	1.27 ± 0.4	52 ± 1.5
		PM	32 ± 1.5	1.5 ± 0.4	50 ± 2
2	Mg 1.98 TiO_2_	DMD [24]	23 ± 5.5 (↓40%)	1.625 ± 0.41	64 ± 3 (↑25%)
		PM	28 ± 1.5 (↓12%)	1.3 ± 0.5	60 ± 1 (↑17%)
3	Mg 2.5 TiO_2_	DMD [24]	21 ± 4 (↓47%)	1.334 ± 0.3	68 ± 1.5 (↑30%)
		PM	25 ± 2.5 (↓18%)	1.3 ± 0.3	64 ± 3 (↑28%)

Note: Percentage change (increase (↑) or decrease (↓)) in the grain size and microhardness values of pure Mg with addition of TiO_2_ NPs synthesized by both DMD and PM technique is represented within brackets.

**Table 4:** Effect of interparticulate spacing between the TiO_2_ NPs in the Mg matrix on the Orowan Strengthening.

Material	Synthesis	Interparticulate Spacing (m)		σ_Orowan_ (MPa)	
		λ_Theoretical_	λ_observed_	Theoretical	Experimental
Mg 1.98 TiO_2_	DMD	4.06 × 10^−8^	(6.3 ± 1) × 10^−7^	62	4 ± 0.5
	PM		(7.35 ± 1) × 10^−7^		3.5 ± 0.5
Mg 2.5 TiO_2_	DMD	3.60 × 10^−8^	(3.82 ± 1.5) × 10^−7^	70	6.6 ± 0.5
	PM		(6.84 ± 2) × 10^−7^		4 ± 0.5

Note: Theoretical and experimental values of interparticulate spacing between the TiO_2_ NPs (λ) are utilized to calculate Orowan strengthening contribution (σ_Orowan_) using Orowan-Ashby equation [25].

### 2.4. Coefficient of Thermal Expansion (CTE)

The CTE values of Mg materials primarily synthesized by both DMD and PM techniques measured in the temperature range of 50–400 °C (Table 1) revealed that the average CTE values of pure Mg decreased with the addition of TiO_2_ NPs. Even though the CTE values of PM synthesized Mg materials were found to be lower, when compared to DMD synthesized Mg-TiO_2_ nanocomposites, the presence of TiO_2_ NPs within the Mg matrix processed by PM technique did not contribute much to the decrease in the CTE values of pure Mg. In the case of DMD synthesized Mg materials, the CTE values of Mg (1.98 and 2.5) TiO_2_ nanocomposites were ~1.48% (26.6 × 10^−6^ /K) and ~1.85% (26.5 × 10^−6^ /K) lower than that of pure Mg (27 × 10^−6^ /K) contributing more to its dimensional stability when compared to that of PM synthesized Mg-TiO_2_ nanocomposites exhibiting a maximum decrease of only ~1.21% (24.42 × 10^−6^ /K) observed with Mg 2.5 vol % TiO_2_ nanocomposite.

### 2.5. Microhardness

The hardness values of both DMD and PM synthesized pure Mg increased with the addition of TiO_2_ NPs (as shown in Table 3). When compared to PM synthesized Mg-TiO_2_ nanocomposites, the hardness values of DMD synthesized Mg nanocomposites were higher and a maximum of ~68 Hv which is ~30% greater than that of pure Mg was observed in the case of DMD synthesized Mg 2.5 vol % TiO_2_ nanocomposite and thereby exhibiting higher constraint to localized plastic deformation.

### 2.6. Tensile Properties

**Figure 4:**
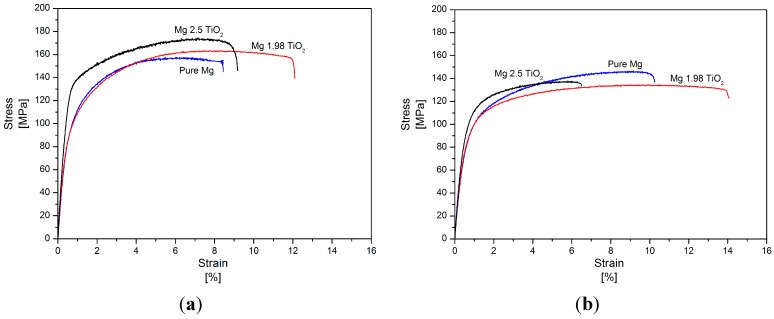
Stress–Strain curves of (**a**) DMD and (**b**) PM synthesized pure Mg and Mg-TiO_2_ nanocomposites during tensile loading.

The room temperature tensile properties of pure Mg and Mg (1.98 and 2.5) vol % TiO_2_ nanocomposites synthesized by both DMD and PM techniques and their representative stress-strain curves are shown in Table 5 and Figure 4, respectively. Among the synthesized Mg materials, PM synthesized Mg 1.98 vol % TiO_2_ nanocomposite exhibited the maximum tensile fracture strain of ~14.5% which is ~55% greater than that of pure Mg. With further addition of TiO_2_ NPs (2.5 vol %), significant decrease in the tensile fracture strain of pure Mg by ~30% was observed. When compared to pure Mg, PM synthesized Mg nanocomposites further exhibited inappreciable changes in the values of 0.2% tensile yield strength (0.2% TYS) and decrease in ultimate tensile strength (UTS) by ~5%. But, in the case of DMD synthesized Mg-TiO_2_ nanocomposites, increase in the 0.2% TYS and UTS of pure Mg with the addition of TiO_2_ NPs was observed exhibiting a maximum increase of ~37% and ~9%, respectively, for Mg 2.5 vol % TiO_2_ nanocomposite [24]. Among the DMD synthesized Mg materials, Mg 1.98 vol % TiO_2_ nanocomposite exhibited the maximum tensile fracture strain of ~11.5% which is ~49% greater than that pure Mg [24]. With further addition of TiO_2_ NPs (2.5 vol %), the tensile fracture strain of DMD synthesized pure Mg decreased to ~10% [24]. The energy absorbed (EA) until failure under tensile loading of both DMD and PM synthesized Mg nanocomposites.

Increased with 1.98 vol % TiO_2_ NPs exhibiting a maximum of ~18 MJ/m^3^ and ~17.5MJ/m^3^, respectively. Further addition of TiO_2_ NPs leads to decrease in the values of EA which is due to poor ductility exhibited by Mg 2.5 vol % TiO_2_ nanocomposites.

**Table 5:** Results of room temperature tensile testing.

Material	Synthesis	0.2% TYS (MPa)	UTS (MPa)	Fracture Strain (%)	Energy Absorbed (MJ/m^3^)
Pure Mg	DMD [24]	92 ± 5	156 ± 6	8.2 ± 0.2	11 ± 1
	PM	89 ± 4.5	142 ± 6	10 ± 0.3	12 ± 1
Mg 1.98 TiO_2_	DMD [24]	102 ± 3 (↑8%)	165.5 ± 3 (↑4%)	11.5 ± 1 (↑49%)	18 ± 0.7 (↑55.8%)
	PM	88 ± 10	132 ± 8 (↓5%)	14.5 ± 1 (↑55%)	17.5 ± 0 (↑35%)
Mg 2.5 TiO_2_	DMD [24]	124 ± 8.8 (↑37%)	170 ± 6 (↑9%)	10 ± 1 (↑34%)	16 ± 2 (↑50%)
	PM	91.1 ± 5 (↑3%)	134 ± 7 (↓5%)	6 ± 1 (↓30%)	8.6 ± 0.6 (↓29%)

Note: Percentage changes (increase (↑) /decrease (↓)) in the tensile properties of the DMD and PM synthesized pure Mg with addition of TiO_2_ NPs are mentioned within brackets.

### 2.7. Compression Properties

**Figure 5:**
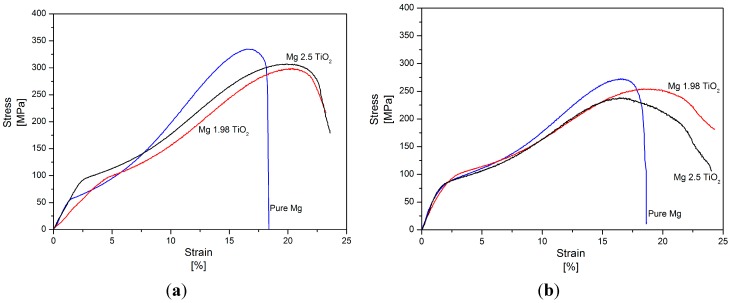
Stress–Strain curves of (**a**) DMD and (**b**) PM synthesized pure Mg and Mg-TiO_2_ nanocomposites during compressive loading.

The room temperature compression properties of DMD and PM synthesized Mg (1.98 and 2.5) vol % TiO_2_ nanocomposites and their representative stress-strain curves are shown in Table 6 and Figure 5, respectively. In the case of PM synthesized Mg 1.98 vol % TiO_2_ nanocomposites, the 0.2% compressive yield strength (0.2% CYS) and compressive fracture strain of pure Mg increased to ~90 MPa and ~25%, respectively which are ~18% and ~26% greater than that of pure Mg. With further addition of TiO_2_ NPs (2.5 vol %), the 0.2% CYS and fracture strain decreased to ~81 MPa and ~24%, respectively. But, in the case of DMD synthesized Mg-TiO_2_ nanocomposites, both the 0.2% CYS and fracture strain increased with the addition of TiO_2_ NPs with a maximum increase of ~83% (~101 MPa) and 33% (~22%), respectively was observed for 2.5 vol % TiO_2_ NPs addition. But, the ultimate compressive strength (UCS) of both PM and DMD synthesized Mg-TiO_2_ nanocomposites was found to be lower than that of synthesized pure Mg. The energy absorbed (EA) until failure under compression loading of both DMD and PM synthesized Mg nanocomposites increased with the addition of TiO_2_ NPs and among the synthesized Mg materials PM synthesized Mg 1.98 vol % TiO_2_ nanocomposites exhibited the maximum EA value of ~46 MJ/m^3^ which is ~37% greater than that of pure Mg.

**Table 6:** Results of room temperature compression testing.

Material	Synthesis	0.2% CYS (MPa)	UCS (MPa)	Fracture Strain (%)	Energy Absorbed (MJ/m^3^)
Pure Mg	DMD [24]	57 ± 3	332 ± 10	18 ± 0	34 ± 2
	PM	76 ± 2	275 ± 4	20 ± 1.5	34 ± 4
Mg 1.98 TiO_2_	DMD [24]	88.3 ± 1 (↑49%)	297 ± 1 (↓13%)	21.9 ± 1 (↑27%)	42 ± 0.5 (↑17%)
	PM	89.9 ± 2 (↑18%)	245 ± 8 (↓9%)	25 ± 2 (↑26%)	46 ± 6 (↑37%)
Mg 2.5 TiO_2_	DMD [24]	101 ± 9 (↑83%)	305.5 ± 11 (↓7%)	22 ± 2 (↑33%)	43 ± 4 (↑31%)
	PM	81 ± 0.6 (↑6)	233 ± 6 (↓14%)	24 ± 2 (↑21%)	37 ± 3 (↑5%)

Note: Percentage change (increase (↑)/decrease (↓)) in the compressive properties of the DMD and PM synthesized pure Mg with addition of TiO_2_ NPs are mentioned within brackets.

## 3. Discussion

### 3.1. Microstructural Characteristics

Microstructural characterization of synthesized Mg-TiO_2_ nanocomposites is discussed in terms of (a) grain size and (b) distribution of reinforcement through measurement of interparticulate spacing. During recrystallization, the presence of second phase ultrafine reinforcements inhibits grain growth more significantly when compared to the larger reinforcements [26]. The grain size of pure Mg was found to decrease with the addition of TiO_2_ NPs suggesting: (a) the ability of ultrafine TiO_2_ NPs to effectively act as nucleation site and (b) inhibiting grain growth by inducing grain boundary pinning. The fundamental principle behind the ability of fine inclusions within the metal matrix to nucleate recrystallized grains and to inhibit grain growth has been established already [27,28]. Reasonable distribution of reinforcements within the metal matrix is possible when a large deformation load is applied in secondary processing [29]. The interparticulate spacing between the TiO_2_ NPs within the Mg matrix measured for the synthesized Mg-TiO_2_ nanocomposites indicated more effective distribution in the case of DMD synthesized Mg nanocomposites (Figure 3). The initial distribution of NPs in PM processed Mg-TiO_2_ nanocomposites depend only on the blending parameters whereas, in the case of DMD, it depends on: (a) arrangement of TiO_2_ NPs and Mg turnings within the crucible prior to heating, (b) vigorous stirring [30] with judicial selection of stirring time and stirring speed based on the density difference between the Mg and reinforcement and (c) disintegration of the composite slurry by argon jets and subsequent deposition in the metallic mould.

The effects of TiO_2_ NPs and synthesis techniques on the crystallographic orientation of pure Mg were analyzed using X-ray diffraction (XRD) studies. For XRD studies along the transverse direction of Mg-TiO_2_ nanocomposite samples, dominance of prismatic intensity was observed indicating that any of the prismatic plane is perpendicular to the extrusion direction [20,21,25]. Along the longitudinal direction, the dominance of basal plane intensity in the extruded pure Mg samples was observed indicating that most of the basal planes are parallel to the extrusion direction, which is commonly found in wrought Mg materials [20,21,31]. With addition of 1.98 vol % TiO_2_ NPs to pure Mg, irrespective of the synthesis technique, the basal plane intensity of pure Mg was found to decrease indicating that the basal planes are no longer parallel to the extrusion direction. But with further addition of TiO_2_ NPs, when compared to that of Mg 1.98 vol % TiO_2_ nanocomposites, the basal intensity of pure Mg was found to increase and exhibiting a strong basal texture in the case of DMD synthesized Mg 2.5 vol % TiO_2_ nanocomposite (*I*_basal_/*I*_max_ = 1). It is found that, irrespective of the synthesis technique, critical amount of TiO_2_ NPs (1.98 vol %) contribute significantly to the weakening of basal texture of pure Mg.

### 3.2. Mechanical Behavior

#### 3.2.1. Hardness

The hardness value of pure Mg was found to increase with the addition of TiO_2_ NPs observed in the case of both DMD and PM techniques. This increase in the microhardness values may be attributed to: (a) uniform distribution of TiO_2_ NPs with minimal agglomeration within the Mg matrix, (b) higher constraint to localized plastic deformation due to the presence of high hardness TiO_2_ NPs (700 Hv) and (c) reduced grain size. Among the synthesized Mg materials, DMD synthesized Mg 2.5 vol % TiO_2_ nanocomposite exhibited higher hardness values of ~68 Hv due to the combination of more finer grains and relatively more uniform distribution of TiO_2_ NPs within the Mg matrix.

#### 3.2.2. Tensile Properties

Among the synthesized Mg materials, DMD synthesized Mg 2.5 vol % TiO_2_ nanocomposite exhibited the maximum 0.2% TYS of ~124 MPa which is ~37% greater than that of pure Mg. Whereas, in the case of PM synthesized Mg materials, the 0.2% TYS of pure Mg marginally increased with Mg 2.5 vol % TiO_2_ exhibiting 0.2%TYS of ~91 MPa which is ~3% greater than that of pure Mg. The mechanisms contributing to the strength of the particulate reinforced metal matrix nanocomposites (MMNCs) (as discussed under Section 1) and the effects of particulate size, amount of particulates added to the matrix and synthesis methodologies on the strengthening mechanisms are discussed for better understanding on the tensile behavior of the synthesized Mg-TiO_2_ MMNCs.

##### Orowan Strengthening

Orowan strengthening is due to the resistance offered by the ultrafine particulates to the dislocation movement by formation of dislocation loops around the particulates. These dislocation loops possess high work hardening rates and thereby assist in strengthening of MMNCs. The strength improvement due to Orowan effect of particulate reinforcement within the Mg matrix is given by Orowan-Ashby equation as shown in Equation (1) [25]:

(1) $${\text{σ}}_{Orowan}=\frac{0.13\text{Gb}}{\text{λ}}\ln\frac{r\;}{b}$$

where, *G* is the shear modulus of Mg (17.3 GPa) [32], b is the burgers vector of Mg (3.21 × 10^−10^ m) [33], *r* and *d*_p_ are the average radius and diameter of NPs, respectively. The interparticulate spacing (λ) between the NPs within the Mg metal matrix is given by Equation (2) [34,35]:

(2) $$\text{λ}=d_{p\;}\left[{\left(\frac{1}{2\;V_{p}\;}\right)}^{\frac{1}{3}}-1\right]$$

where, *V*_p_ is the volume fraction addition of TiO_2_ NPs.

The design of Mg matrix composites based on the theoretical contribution of Orowan strengthening varying with (a) size of the reinforcements such as nano (10 to 100 nm), sub-micron (0.1 and 0.5 μm) and micron sized (1 μm) and (b) volume fraction of the reinforcement particulates and is shown in Table 7. Considering the cost of the NPs and the effectiveness of the available synthesis methodologies to uniformly disperse the NPs within the Mg metal matrix, a critical threshold of 2.5 vol % is considered in the case of Mg MMNCs. From Table 7, for a constant volume fraction, the contribution of Orowan strengthening decreases with the increase in the size of the particulates. For example, considering a constant volume fraction of 2.5 vol %, the Orowan strengthening contribution for particulate size of ~1 μm is almost negligible whereas strength as high as ~147 MPa is theoretically predicted for particulate size of 10 nm. In the case of Mg matrix microcomposites, maximum strength of ~65 MPa is predicted with particulate size and quantity of 0.5 μm, 40 vol %, respectively. However, presence of high volume fraction reinforcements significantly contributes to the density of the Mg materials which pose a major limitation especially targeting towards synthesizing light-weight materials.

For activation of Orowan strengthening, synthesis methodologies play a vital role in controlling the interparticulate spacing for avoiding clustering of particulate reinforcements within the metal matrix.

Table 7 shows the theoretically predicted interparticulate spacing for varying the particulate size and quantity of reinforcements. Interparticulate spacing between the particulates was found to decrease with increasing volume fraction and decreasing particulate size. It is difficult to control the interparticulate spacing in the case of 40 vol % reinforced Mg microcomposite as well as 2.5 vol % reinforced Mg MMNCs which are ~3.86 × 10^−8^ m and ~1.78 × 10^−8^ m (Table 7) for 0.5 μm and 10 nm sized particulates, respectively. Table 4 shows the theoretically predicted and experimentally observed interparticulate spacing of TiO_2_ NPs within the synthesized Mg-TiO_2_ nanocomposites. It is observed that by utilizing the adopted synthesis techniques (DMD and PM), the interparticulate spacing observed through microstructural characterization of the nanocomposites is not sufficient to contribute to the Orowan strengthening.

##### Hall-Petch Strengthening

Hall-Petch strengthening (σ*_Hall-Petch_*) effect relates the effect of grain size on the strength of the material. Grain size is inversely proportional to the strength of the material: as the grain size decreases, the strength of the material increases. The following equation describes the Hall-Petch equation:

(3) $${\text{σ}}_{Hall-Petch}=KD^{-0.5}$$

where *K* is the Hall-Petch coefficient of Mg (280 MPa·μm^1/2^) and *D* is the average grain size of synthesized Mg composites. Further, the relationship between reinforcement particulate size, volume fraction of particulates and grain size of composites is given by Zener equation Equation (4) [36]:

(4) $$d_{m}=\;\frac{4\;\text{α}\;d_{p}}{3\;v_{p}}$$

where α is proportionality constant, and *d_m_* is the grain size of the metal matrix. The grain size of MMNCs is found to decrease with: (a) decrease in the particulate size and (b) higher volume fraction addition of NPs. In the present study, reduction in the grain size of both DMD and PM synthesized pure Mg with the addition of (1.98 and 2.5) vol % TiO_2_ NPs was observed (Table 3). The contribution of Hall-Petch strengthening on the TYS of Mg nanocomposites is found by utilizing Equation (3) and it is observed that among the synthesized Mg materials, DMD synthesized Mg (1.98 and 2.5) vol % TiO_2_ nanocomposites exhibited higher σ*_Hall-Petch_* of ~58 MPa and ~61 MPa, respectively due to their finer grain sizes.

##### Strengthening Due to Load Bearing

The strengthening contribution due to load bearing (σ*_load transfer_*) of equiaxed particulate reinforcements on the TYS of composites which is attained by transferring of applied load to the particulates within the metal matrix is given by Equation (5) [37]:

(5) $${\text{σ}}_{LT}=0.5\;v_{p\;}{\text{σ}}_{Mg}$$

where σ_Mg_ is the experimental 0.2% TYS of pure Mg. The contribution of load transfer mainly depends on the quantity of reinforcement available within the metal matrix. For example, considering an experimental 0.2% TYS of 90 MPa (present study), it is observed that, for 2.5 vol % of addition of reinforcements to Mg matrix, only ~1 MPa increase due to σ*_LT_* is predicted. Whereas, if 40 vol % of particulate reinforcement is added to pure Mg, for 0.2% TYS of 90 MPa, σ*_LT_* contribution of 18 MPa is predicted. As discussed under “Orowan Strengthening”, the cost of NPs and effectiveness of synthesis methodology to uniformly disperse NPs within the metal matrix restricts synthesizing high volume fraction MMNCs and thereby for modeling of MMNCs containing NPs of low volume fraction, strengthening contribution due to load bearing is negligible.

##### Forest Strengthening

The strengthening contribution due to forest strengthening (σ_CTE_) or mismatch between the CTE values of reinforcement particulates and Mg matrix leads to generation of dislocations nearby the particulates and thereby contributes to the increase in dislocation density and strength of the composite. The presence of high dislocation density near the interface between the matrix and reinforcement particulates generated due to CTE mismatch has been experimentally observed [37,38]. Forest strengthening effect generated due to CTE mismatch can be described by the following Equations (6) and (7):

(6) $${\text{σ}}_{CTE}=A\cdot M\cdot G\cdot b\cdot {{\text{ρ}}_{th}}^{0.5}$$

(7) $${\text{ρ}}_{th}=\frac{12\sqrt{2}\cdot \Delta \text{α}\cdot \Delta T\cdot f}{b\cdot d\cdot \left(1-f\right)}$$

where *A* is a constant characterizing the transparency of dislocation forest for basal-basal interaction in Mg (0.2) [33], ρ_th_ is the dislocation density, Δα is the difference in the CTE values between the matrix (Pure Mg) and reinforcement, Δ*T* is the temperature excursion which is chosen to be 250 K (for all the nanocomposites) assuming that the dislocation generation begins at 550 K corresponding to a stress-free homologous temperature of 0.6 [33]. From Equations (6) and (7), theoretically, the forest strengthening contribution (σ_CTE_) value is found to increase with the addition of ultrafine reinforcements of higher volume fraction. Experimentally, Vogt *et al.* [39] observed that when compared to microcomposites, forest strengthening was found negligible in the case of MMNCs synthesized by PM process by plotting the true stress-strain curves of MMNCs specimens under various heat treatment and quenching conditions. It is found to be low for particulate size less than 80 nm due to the limitations of synthesis methodologies to uniformly disperse ultrafine NPs [40]. Further, Chawla *et al.* [41,42] reported that forest strengthening can be realized only with higher volume fraction addition (greater than 10 vol %). In the present study, the contribution of forest strengthening, theoretically calculated by utilizing Equations (6) and (7), on the TYS of pure Mg was found to be ~115 MPa and ~130 MPa for Mg (1.98 and 2.5) vol % TiO_2_ nanocomposites, respectively.

##### Taylor Strengthening

The strengthening contribution due to Taylor strengthening (σ_EM_) or mismatch between the elastic modulus values of the reinforcement particulates and the metal matrix leading to the formation of geometrically necessary dislocations (GND) due to straining or presence of external load is given by Equations (8) and (9) [43]:

(8) $${\text{σ}}_{EM}=\sqrt{3}\cdot \text{α}\cdot G\cdot b\cdot \surd {\text{ρ}}_{EM}$$

(9) $${\text{ρ}}_{EM}=\;\frac{6\;V_{p}}{\text{π}d^{3\;}}$$

where α is a constant (0.5), ρ*_EM_* is density of dislocations due to modulus mismatch and ξ is uniform deformation or strain. Theoretically, the strengthening contribution due to Taylor strengthening is found to increase with the presence of ultrafine particulates of larger volume fraction. During extrusion, it is observed that dislocations produced by work hardening effects accumulate in the coarse grain regions (on the order of 1000 μm) and density of dislocation in fine grained regions is insignificant [44,45]. Further, in the case of Mg MMNCs, when compared to the contributions of other strengthening mechanisms, Taylor strengthening is considered negligible.

For theoretically predicting the tensile yield strength (TYS) of the MMNCs, several models such as: (1) summation of strengthening [46], (2) Zhang and Chen model [47,48], and (3) modified Clyne models [20,43] have been utilized. In the summation of strengthening contributions model, the individual contributions of the strengthening mechanisms are simply added along with the TYS of the Mg matrix to get the overall strength of the composite assuming that each strengthening mechanism behaves independently [43]. Summation of strengthening contributions (σ*_summation_*) model is given by Equation (10):

(10) $${\text{σ}}_{summation}={\text{σ}}_{Mg}+{\text{σ}}_{Orowan}+{\text{σ}}_{Hall-Petch}+{\text{σ}}_{CTE}+{\text{σ}}_{EM}+{\text{σ}}_{LT}$$

Modified Clyne model was developed by Sanatay-Zadeh [43]. In this model, the root of sum of squares of contributions of different strengthening mechanisms is calculated and finally added to 0.2% TYS (σ*_Mg_*). The modified Clyne (σ*_modified Clyne_*) model is given by the following Equations (11) and (12):

(11) $${\text{σ}}_{modified\;Clyne}={\text{σ}}_{Mg}+\Delta \text{σ}$$

(12) $$\Delta \text{σ}=\sqrt{{{\text{σ}}_{Orowan}}^{2}+{{\text{σ}}_{Hall-Petch}}^{2}+{{\text{σ}}_{CTE}}^{2}+{{\text{σ}}_{EM}}^{2}+{{\text{σ}}_{LT}}^{2}}$$

**Table 7:** Designing of Mg composites based on contribution of Orowan strengthening calculated by using Orowan- Ashby equation [25].

Volume Fraction (in %)	Orowan Stress σ_orowan_ (in MPa) and Interparticulate Spacing λ (in m)	Reinforcement Particulate Size															
		In (nm)													In (μm)		
		10	20	21 *	25	30	40	50	60	70	75	80	90	100	0.5	0.75	1
0.58	σ_orowan_	73.66	36.83	35.08	29.46	24.55	18.41	14.73	12.27	10.52	9.82	9.20	8.18	7.36	1.47	1.00	0.74
	λ (× 10^−8^)	3.41	6.83	7.17	8.54	10.3	33.7	17.0	20.5	23.9	25.6	27.3	30.8	31.4	170	256	341
0.97	σ_orowan_	92.50	46.25	44.05	37.00	30.83	23.12	18.50	15.41	13.21	12.33	11.56	10.27	9.25	1.85	1.23	0.92
	λ (× 10^−8^)	2.72	5.44	5.71	6.80	8.16	10.8	13.6	16.3	19.0	20.4	21.8	24.4	27.2	136	204	272
1.50	σ_orowan_	113.50	56.74	54.04	45.39	37.83	28.37	22.69	18.91	16.21	15.13	14.18	12.61	11.34	2.26	1.51	1.13
	λ (× 10^−8^)	2.21	4.43	4.65	5.54	6.65	8.87	11.0	13.3	15.5	16.6	17.7	19.9	22.1	110	166	221
1.98	σ_orowan_	130.19	65.09	62.00	52.07	43.39	32.54	26.03	21.69	18.59	17.35	16.27	14.46	13.01	2.60	1.73	1.30
	λ (× 10^−8^)	1.93	3.87	4.06	4.83	5.80	7.73	9.66	11.6	13.5	14.5	15.4	17.4	19.3	96.6	145	193
2.50	σ_orowan_	146.85	73.42	69.93	58.74	48.95	36.71	29.37	24.48	20.97	19.58	18.35	16.31	14.69	2.93	2.00	1.46
	λ (× 10^−8^)	1.71	3.42	3.60	4.28	5.14	6.85	8.57	10.2	12.0	12.8	13.7	15.4	17.1	85.7	128	171
10.00	σ_orowan_	**Not Applicable**													7.09	4.72	3.54
	λ (× 10^−8^)														35.4	53.2	71.0
20.00	σ_orowan_														14.09	9.39	7.04
	λ (× 10^−8^)														17.8	26.7	35.7
30.00	σ_orowan_														27.12	18.08	13.56
	λ (× 10^−8^)														9.28	1.39	18.5
40.00	σ_orowan_														65.21	43.47	32.60
	λ (× 10^−8^)														3.86	5.79	7.72

Note: * Present Study.

Zhang and Chen [34] derived an empirical model for predicting the TYS of Mg MMNCs by studying the effects of volume fraction and size of alumina NPs without considering the contribution of Hall-Petch strengthening. Zhang and Chen σ*_ZC_* model is given by the following Equation (13):

(13) $$\sigma_{ZC}=(1+0.5\;v_{p})({\text{σ}}_{Mg}+{\text{σ}}_{Orowan}+{\text{σ}}_{CTE}+\frac{{\text{σ}}_{Orowan}\times {\text{σ}}_{CTE}}{{\text{σ}}_{Mg}})$$

**Figure 6:**
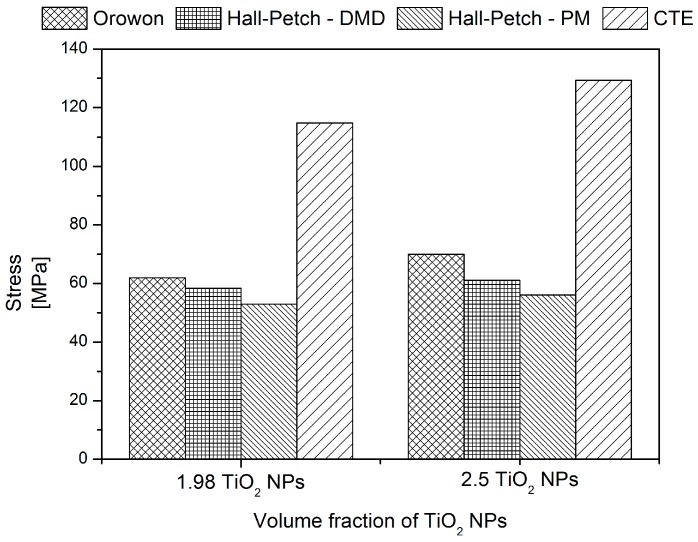
Individual contribution of strengthening mechanisms on the TYS of DMD and PM synthesized Mg/TiO_2_ nanocomposites.

In the present study, pure Mg is reinforced with (1.98 and 2.5) vol % TiO_2_ with TiO_2_ particulates of size ~21 nm. The contributions of σ*_LT_* and σ*_EM_* are considered insignificant or negligible. Further, σ*_summation_* and σ*_modified Clyne_* are calculated for DMD and PM synthesized Mg-TiO_2_ nanocomposites with and without considering the contribution of Forest strengthening (σ*_CTE_*). Theoretically, the individual contributions of Orowan (σ*_Orowan_*), Hall-Petch (σ*_Hall-Petch_*) and Forest strengthening (σ*_CTE_*) are calculated using the Equations (1) to (7) and are shown in Figure 6 and Table 8. Among the strengthening mechanisms theoretically calculated for Mg-TiO_2_ nanocomposites, the contribution of Forest strengthening (σ*_CTE_*) is found to be the maximum with ~115 MPa and ~130 MPa for Mg (1.98 and 2.5) vol % TiO_2_ NPs, respectively. From microstructural characterization, the contribution of Orowan strengthening calculated by using the observed λ values for both DMD and PM processed nanocomposites indicate that the strengthening is almost negligible. Owing to the insignificance of Orowan strengthening, both the summation of strengthening and modified Clyne models reduce to the below Equation (14) which indicates that the TYS of Mg-TiO_2_ MMNCs predominantly depends only on the σ*_Hall-Petch_*:

(14) $$\text{σ}={\text{σ}}_{Mg}+\;{\text{σ}}_{Hall-Petch}$$

**Figure 7:**
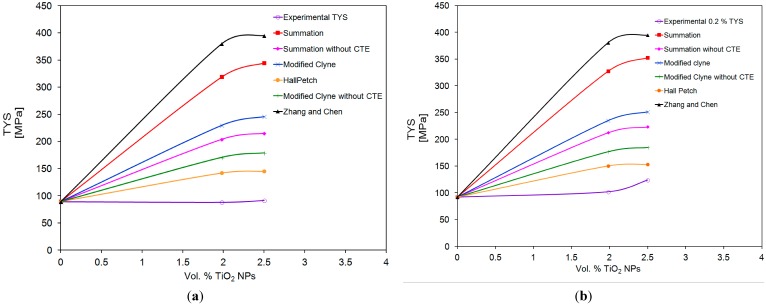
Experimental and Predicted TYS values of DMD and PM synthesized Mg/TiO_2_ nanocomposites.

The theoretical TYS values of the DMD and PM synthesized Mg-TiO_2_ nanocomposites computed utilizing the existing mathematical models in the literature (Equations (10) to (13)) and Hall Petch model (Equation 14) are compared with the experimentally observed 0.2% TYS values of Mg (1.98 and 2.5) vol % TiO_2_ nanocomposites, respectively and is shown in Figure 7 and Table 8. The experimental 0.2% TYS values are found to be in close agreement with the predicted TYS considering only Hall-Petch strengthening Equations (14).

**Table 8:** Contribution of different strengthening mechanisms and predicted values of tensile yield strength of DMD and PM synthesized Mg/TiO_2_ nanocomposites using analytical models.

Material	Synthesis	0.2% TYS Experimental (MPa)	σ_Orowan_ (MPa)	σ_Hall-Petch_ (MPa)	σ_CTE_ (MPa)	σ_Summation_ (MPa)		σ_Modified Clyne_ (MPa)		σ_ZC_ (MPa)	σ_Mg +_ σ_Hall-Petch_ (MPa)
						With σ_CTE_	Without σ_CTE_	With σ_CTE_	Without σ_CTE_		
Pure Mg	DMD	92 ± 5	–	–	–	–	–	–	–	–	–
	PM	89 ± 4.5									
Mg 1.98 TiO_2_	DMD	102 ± 3	62	58.3	114.82	327.1	212.2	234.9	177.0	349.61	150.3
	PM	88 ± 10		52.9		318.7	203.88	230.0	170.5	380.041	142.0
Mg 2.5 TiO_2_	DMD	124 ± 8.8	70	61.1	129.36	352.4	223.04	251.2	185.0	394.65	153.1
	PM	91.1 ± 5		56.0		344.3	215.00	246.4	178.6	394.98	145.0

**Figure 8:**
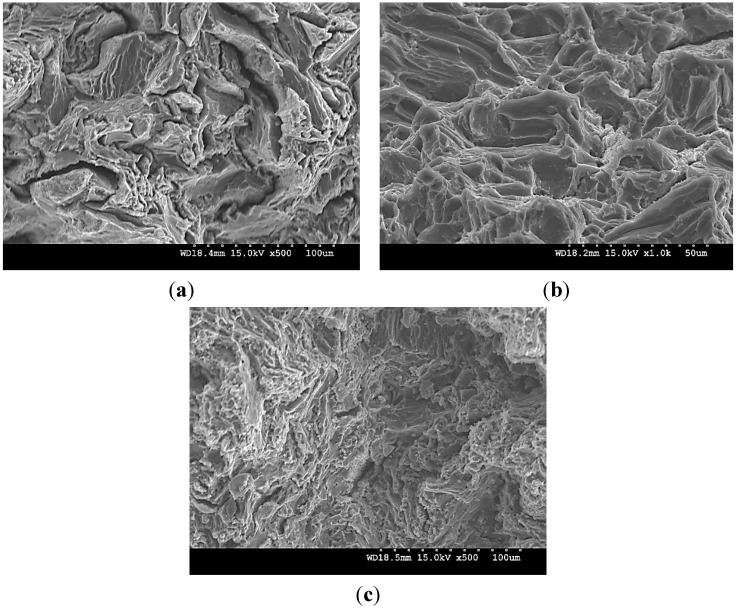
Fractographs after tensile loading of PM synthesized (**a**) pure Mg, (**b**) Mg 1.98 TiO_2_ and (**c**) Mg 2.5 TiO_2_.

Limited increase in the 0.2% TYS of pure Mg (particularly with 1.98 vol % TiO_2_) and changes in its tensile fracture strain with the addition of TiO_2_ NPs is due to: (a) induced textural changes of pure Mg due to the presence of NPs (Table 2 and Figure 1) [20,21,24,49] and (b) distribution or agglomeration of TiO_2_ NPs within the Mg matrix. From the XRD studies (Table 2), the ratio of *I*_basal_/*I*_max_ value measured along longitudinal sections of DMD and PM synthesized Mg 1.98 vol % TiO_2_ nanocomposite samples are ~0.751 and ~0.655, respectively. The decrease in the basal plane intensity of pure Mg indicates non-basal cross-slip activation in PM synthesized pure Mg due to the presence of 1.98 vol % TiO_2_ NPs which is evident from its maximum tensile fracture strain value of ~14.5% when compared to that of DMD synthesized Mg 1.98 vol % TiO_2_ nanocomposite (~11.5%). Relatively lower tensile fracture strain in DMD synthesized Mg 2.5 vol % TiO_2_ nanocomposite is due to the dominance of basal texture with increase in the ratio of *I*/*I*_max_ corresponding to the basal plane intensity (*I*/*I*_max_ = 1) measured along its longitudinal section. Significant decrease in the tensile fracture strain of PM synthesized Mg 2.5 vol % TiO_2_ nanocomposite is due to the combined effect of (a) visible agglomeration sites of TiO_2_ NPs within Mg matrix and (b) increase in the ratio of (*I*_basal_/*I*_max_) measured along the longitudinal sections to 0.844. The mathematical models available in the literature over predict the TYS of Mg MNNCs. For effective design of Mg MMNCs, texture effects and effectiveness of synthesis methodologies to disperse the NPs within the Mg metal matrix through careful microstructural investigation may have to be considered for more realistic prediction of TYS. The mode of fracture under tensile loading of DMD synthesized pure Mg and Mg-TiO_2_ nanocomposites with representative fractographs are discussed already [24]. For all the DMD synthesized pure Mg and nanocomposite samples, typical cleavage mode of fracture was observed [24]. Figure 8 shows the tensile fracture surfaces of PM synthesized pure Mg and Mg (1.98 and 2.5) vol % TiO_2_ nanocomposite. Fracture surfaces of PM synthesized pure Mg and Mg 2.5 vol % TiO_2_ samples (Figure 8a,c) indicate microscopically rough features with cleavage steps highlighting brittle failure which is also evident from their tensile fracture strain values of ~10% and 6%, respectively. Whereas, in the case of PM synthesized Mg 1.98 vol % TiO_2_, fractograph indicates mixed mode of failure with dimple like features thereby indicating possibilities of plastic deformation of Mg matrix which is also evident from its high tensile fracture strain value of ~14.5%.

#### 3.2.3. Compressive Properties

**Figure 9:**
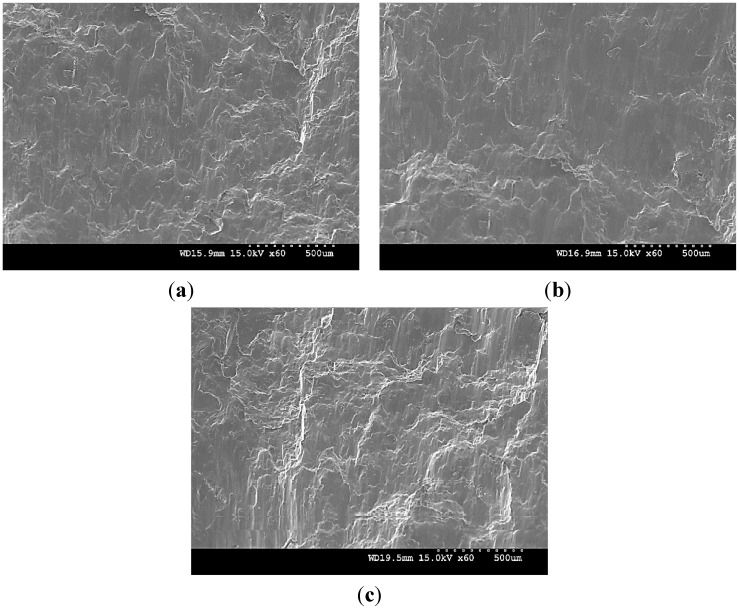
Fractographs after compressive loading of PM synthesized (**a**) pure Mg, (**b**) Mg 1.98 TiO_2_ and (**c**) Mg 2.5 TiO_2_.

The increase in the 0.2% CYS of pure Mg observed with PM synthesized 1.98 vol % TiO_2_ nanocomposites and DMD synthesized Mg-TiO_2_ nanocomposites is due to the significant grain refinement of Mg materials contributing to the Hall-Petch strengthening. When compared to 1.98 vol % TiO_2_ NPs addition, the decrease in the compression properties of PM synthesized pure Mg with addition of 2.5 vol % TiO_2_ NPs is due to the presence of agglomeration sites visible through microstructural analysis. Further, the decrease in the UCS of pure Mg observed in Mg-TiO_2_ nanocomposites may be due to: (a) possible clustering of TiO_2_ NPs within the Mg matrix which is evident from the measured interparticulate spacing values (λ_observed_) between the NPs, (b) absence of Orowan strengthening contribution, (c) poor load bearing capabilities of low volume fraction NPs which is evident from the negligible strengthening contribution due to load bearing of NPs (Equation (5)). The fracture surfaces of DMD synthesized pure Mg [25] and Mg-TiO_2_ nanocomposites are discussed (with representative fractographs after compressive loading). Figure 9 shows the fracture surfaces of the PM synthesized pure Mg and Mg-TiO_2_ nanocomposites under compressive loading. Similar to the DMD synthesized Mg-TiO_2_ nanocomposites, fracture in pure Mg and Mg-TiO_2_ nanocomposite occurred at 45° with respect to the compression loading axis and their representative fractographs indicate presence of shear bands.

Under tensile loading of extruded Mg materials, basal slip is the dominant deformation mechanism and under compression loading, due to the lower critically resolved shear stress (CRSS) requirements to initiate twinning than basal slip, tensile twinning occurs [20,21,24,49]. The directional nature of twinning makes Mg materials to show large anisotropy when deformed under different stress states and initial textures [50,51]. A way to capture the anisotropy is through measuring tensile-compression anisotropy “TCA” value which is σ*_y,t_*/σ*_y,c_*, where σ*_y,t_* and σ*_y,c_* are uniaxial tensile and compressive yield strengths, respectively [52]. The TCA values of PM and DMD synthesized pure Mg and Mg-TiO_2_ nanocomposites are shown in Table 9. The TCA values of both DMD and PM synthesized Mg-TiO_2_ nanocomposites is found to be less than that of pure Mg. Further, minimum TCA values of ~1.17 and ~1.06 in the case of both DMD and PM synthesized Mg 1.98 vol % TiO_2_ nanocomposites exhibiting maximum tensile fracture strain of ~11.5% and ~14.5%, respectively, was observed. This is due to the non-basal cross-slip activation which is further confirmed through XRD studies (Table 2) as discussed under Section 3.1.

**Table 9:** Room temperature Tensile–Compression Asymmetry (TCA) of the synthesized pure M and Mg-TiO_2_ nanocomposites.

Material	Synthesis	σ*_y,t_* (MPa)	σ*_y,c_* (MPa)	TCA
Pure Mg	DMD	92 ± 5	57 ± 3	1.61
	PM	89 ± 4.5	76 ± 2	1.20
Mg 1.98 TiO_2_	DMD	102 ± 3	88.3 ± 1	1.17
	PM	88 ± 10	89.9 ± 2	1.06
Mg 2.5 TiO_2_	DMD	124 ± 8.8	101 ± 9	1.20
	PM	91.1 ± 5	81 ± 0.6	1.13

## 4. Experimental Section

### 4.1. Materials

In the present study, for the base material, >99.9% pure elemental magnesium turnings supplied by ACROS organics (Morris Plains, NJ, USA) was utilized in DMD technique and for blend-press-sinter powder metallurgy technique, Mg powder of ≥98.5% purity with a size range of 60–300 μm supplied by Merck (Darmstadt, Germany) was utilized. For reinforcement phase, for both the synthesis techniques, required amount of pure titanium dioxide TiO_2_ P25 powder of size ~21 nm and purity >99.5% supplied by Sinopharm Chemical Reagent Co., Ltd (Beijing, China) was utilized.

### 4.2. Processing

#### 4.2.1. Primary Processing

Monolithic magnesium and Mg (1.98 and 2.5) vol % TiO_2_ nanocomposites were primarily synthesized by utilizing DMD or disintegrated melt deposition technique (liquid synthesis) and PM or blend-press-sinter powder metallurgy technique (solid synthesis). The procedures involved in DMD techniques are briefly discussed in previous works [20,21,53,54]. The 40 mm diameter ingots obtained from DMD techniques were then machined to a diameter of 36 mm for hot extrusion.

In the case of blend-press-sinter powder metallurgy technique, initially, pure magnesium powder was blended with appropriate amount of TiO_2_ NPs in a RETSCH PM-400 mechanical alloying machine (Haan, Germany) at 200 rpm for 1 h. The homogenized powder mixtures of Mg and reinforcement were then cold compacted at a pressure of ~1000 MPa to form billets of 40 mm in height and 35 mm in diameter. Monolithic magnesium was compacted using the same parameters without blending. Finally, the compacted billets were sintered using hybrid microwave sintering technique [55]. The billets were heated to 640 °C in a 900 W, 2.45 GHz SHARP microwave oven.

#### 4.2.2. Secondary Processing

Before extrusion, the machined ingots and the sintered billets were soaked at 400 °C for 1 h in a constant temperature furnace. Using a 150 T hydraulic press, hot extrusion was carried out at 350 °C die temperature, with an extrusion ratio of 20.25:1 for obtaining rods of 8 mm in diameter. The samples from the extruded rods were used for characterization, as detailed in the next section.

### 4.3. Materials Characterization

#### 4.3.1. Density Measurements

Density of extruded pure Mg and Mg-TiO_2_ nanocomposites in polished condition was measured using Archimedes principle. Three samples from different ends of the extruded rods were accurately weighed in air and then immersed in distilled water. An A&D ER-182A electronic balance with an accuracy of 0.0001 g was used for measuring the weights. Using rule of mixture principle, the theoretical densities of the synthesized Mg materials were calculated. Porosity values of the synthesized Mg materials were calculated utilizing Equation (15):

(15) $$Porosity=\;\frac{{\text{ρ}}_{th}-\;{\text{ρ}}_{exp}}{{\text{ρ}}_{th}-\;{\text{ρ}}_{air}}\;\times 100$$

where ρ*_th_* is the theoretical density (g/cm^3^), ρ*_exp_* is the experimental density in (g/cm^3^) and ρ*_air_* is the density of air (0.001225 g/cm^3^).

#### 4.3.2. X-Ray Diffraction Studies

The extruded pure magnesium and Mg (1.98 and 2.5) vol % TiO_2_ nanocomposite samples were exposed to Cu Kα radiation of wavelength λ = 1.54056 Å with a scan speed of 2 °/min by using an automated Shimadzu lab-X XRD-6000 diffractometer (Kyoto, Japan). The bragg angles and the values of the interplanar spacing, *d*, obtained were subsequently matched with the standard values of Mg, TiO_2_ and related phases. Further, the basal plane orientation of Mg-TiO_2_ nanocomposites was analyzed based on the XRD peaks obtained from experiments carried out in the directions both parallel and perpendicular to the extrusion axis.

#### 4.3.3. Microstructural Characterization

To investigate on TiO_2_ reinforcement distribution, interparticulate spacing between the NPs within the Mg metal matrix and grain size of pure magnesium and Mg-TiO_2_ nanocomposites, the microstructural characterization studies were conducted on metallographically polished extruded samples and a Hitachi S-4300 field emission scanning electron microscope (FESEM) (Tokyo, Japan), an Olympus metallographic optical microscope(Tokyo, Japan) and Scion image analysis software (Sacramento, CA, USA) were utilized. The interparticulate spacing between the NPs within the nanocomposite samples were graphically estimated using the respective FESEM micrographs and by utilizing Scion image analyzing software. For every nanocomposite samples, five micrographs were utilized for more accurate estimation of grain size and interparticulate spacing.

#### 4.3.4. Coefficient of Thermal Expansion

By using a thermo mechanical analysis instrument “INSEIS TMA PT 1000LT”, the coefficient of thermal expansion values of pure magnesium and Mg (1.98 and 2.5) vol % TiO_2_ nanocomposites were determined. Heating rate of 5 °C/min was maintained with an argon flow rate of 0.1 lpm. By using an alumina probe, the displacement of the test samples (each of 5mm length) was measured as a function of temperature (323 K to 623 K).

#### 4.3.5. Microhardness Test

Using a Shimadzu HMV automatic digital microhardness tester (Kyoto, Japan) with a Vickers indenter (square based pyramidal shaped diamond indenter with a phase angle of 136°), the microhardness tests were conducted on flat and metallographically polished specimens. An indenting load of 25 gf for a dwell time of 15 s was used. The testing was performed as per ASTM E384-08 [56].

#### 4.3.6. Tensile Test

In accordance with ASTM E8M-01 [57], the smooth bar tensile properties of pure magnesium and Mg (1.98 and 2.5) vol % TiO_2_ nanocomposites were determined at ambient temperature. The tensile tests were conducted on round tension test specimens of diameter 5 mm and gauge length 25 mm using a fully automated servo-hydraulic mechanical testing machine, MTS-810. The strain rate was set to 1.693 × 10^−4^ s^−1^ and an Instron 2630-100 series extensometer (Singapore) was used to measure the fracture strain. For each composition, five samples were tested to ensure repeatable values.

#### 4.3.7. Compression Test

In accordance with ASTM E9-89a [58], the smooth bar compressive properties of the extruded pure magnesium and Mg (1.98 and 2.5) vol % TiO_2_ samples were determined at ambient temperature, using MTS-810 testing machine with a strain rate of 8.334 × 10^−5^ s^−1^. The test specimens of 8 mm diameter and length to diameter ratio l/*d* ~ 1 were used. For each composition, five samples were tested to ensure repeatable values.

#### 4.3.8. Fracture Behavior

To provide an insight into the various possible fracture mechanisms operating during the tensile and compression loading of the samples, characterization of fractured surfaces of tensile and compression samples were successfully investigated using Hitachi S-4300 FESEM (Tokyo, Japan).

## 5. Conclusions

In the present study, near dense pure Mg and Mg (1.98 and 2.5) vol % TiO_2_ nanocomposites are successfully synthesized using blend-press-sinter technique (solid-phase synthesis) with Mg 2.5 vol % TiO_2_ nanocomposite exhibiting a maximum porosity of 0.32%. The microstructural and mechanical properties of the PM synthesized nanocomposites are compared to that of the DMD (liquid-phase synthesis) synthesized Mg-TiO_2_ nanocomposites and the following primary conclusions are made:

1. Grain size of both DMD and PM synthesized pure Mg decreases with the addition of TiO_2_ NPs contributing to the Hall-Petch strengthening. For the same volume fraction addition of TiO_2_ NPs, when compared to PM technique, grain refinement in DMD synthesized pure Mg is found to be more significant with ~40% and ~47% for (1.98 and 2.5) vol % TiO_2_ NPs addition, respectively.
2. Presence of TiO_2_ NPs in pure Mg increases the microhardness values with DMD synthesized pure Mg exhibiting a maximum improvement in the hardness values of ~25% and ~30% for (1.98 and 2.5) vol % TiO_2_ NPs additions, respectively.
3. For designing of Mg MMNCs, critical selection on particulate size and volume fraction addition of NPs along with effectiveness of the adopted synthesis methodology to activate Orowan strengthening through achieving more uniform distribution of NPs has to be studied. The results of microstructural characterization for investigating the minimum interparticulate spacing between the TiO_2_ NPs indicate that DMD synthesis is relatively more effective in the dispersion of TiO_2_ NPs with minimal agglomeration of TiO_2_ NPs. However, for both the synthesis techniques, the contribution of Orowan strengthening theoretically calculated utilizing the observed interparticulate spacing within the Mg matrix of the synthesized Mg-TiO_2_ nanocomposite is found to be insignificant indicating absence of Orowan strengthening in Mg-TiO_2_ nanocomposites.
4. Among the mechanisms contributing to the strength of Mg MMNCs, Hall Petch strengthening is found to be the only significant mechanism with ${\text{σ}}_{Mg}+\;{\text{σ}}_{Hall-Petch}$ (Equation (14)) (Hall-Petch) model in close agreement with the experimental 0.2% TYS of both DMD and PM synthesized nanocomposites.
5. Under tensile loading, the strength improvement was more noticeable in Mg-TiO_2_ nanocomposites synthesized through liquid-phase synthesis (DMD technique). PM synthesized Mg 1.98 vol % TiO_2_ exhibited a maximum tensile fracture strain (~14.5%) along with inappreciable changes in the 0.2% TYS. When compared to DMD synthesized Mg-TiO_2_ nanocomposites, the increase in the tensile fracture strain of PM synthesized Mg 1.98 vol % TiO_2_ is due to the activation of non-basal cross-slip which is confirmed from the XRD studies and TCA values.
6. Under compression loading, the 0.2% CYS of PM synthesized pure Mg was found to increase with the addition of 1.98 vol % TiO_2_ NPs by ~8% (~90 MPa) and further addition of 2.5 vol % lead to decrease (~81 MPa) in the 0.2% CYS. The UCS of both the DMD and PM synthesized pure Mg decreased with the addition of TiO_2_ NPs. Maximum fracture strain of ~25% and energy absorbed until fracture of ~46 MJ/m^3^ was observed in the case of PM synthesized Mg 1.98 vol % TiO_2_ nanocomposite.
7. For Mg-TiO_2_ composite system, powder metallurgy route is more effective in minimizing the tensile-compression asymmetry (TCA) with Mg 1.98 vol % TiO_2_ exhibiting TCA value as low as 1.06 for Mg 1.98 vol % TiO_2_ nanocomposite.
